# Pharmacokinetics and Pharmacodynamics of Nemonoxacin in a Neutropenic Murine Lung Infection Model Against *Streptococcus Pneumoniae*

**DOI:** 10.3389/fphar.2021.658558

**Published:** 2021-05-04

**Authors:** Xin Li, Yuancheng Chen, Xiaoyong Xu, Yi Li, Yaxin Fan, Xiaofen Liu, Xingchen Bian, Hailan Wu, Xu Zhao, Meiqing Feng, Beining Guo, Jing Zhang

**Affiliations:** ^1^ Institute of Antibiotics, Huashan Hospital, Fudan University, Shanghai, China; ^2^ Key Laboratory of Clinical Pharmacology of Antibiotics, National Health and Family Planning Commission, Shanghai, China; ^3^ National Clinical Research Center for Aging and Medicine, Huashan Hospital, Fudan University, Shanghai, China; ^4^ Phase I Unit, Huashan Hospital, Fudan University, Shanghai, China; ^5^ Department of Biological Medicines & Shanghai Engineering Research Center of Immunotherapeutics, School of Pharmacy, Fudan University, Shanghai, China

**Keywords:** nemonoxacin, pharmacokinetics/pharmacodynamics, *streptococcus pneumoniae*, neutropenic murine lung infection model, breakpoint

## Abstract

Nemonoxacin, a novel nonfluorinated quinolone for the treatment of community-acquired pneumonia. We reported the pharmacokinetic/pharmacodynamic (PK/PD) targets and PK/PD breakpoints of nemonoxacin against *Streptococcus pneumoniae* using a neutropenic murine lung infection model. Single-dose PK analysis after subcutaneous administration of nemonoxacin at doses from 2.5 to 80 mg/kg showed maximum plasma concentration (C_max_) 0.56–7.32 mg/L, area under the concentration-time curve from 0 to 24 h (AUC_0-24_) 0.67–26.10 mg·h/L, and elimination half-life (T_1/2_) 0.8–1.4 h. The epithelial lining fluid (ELF) penetration ratio of total drug was 1.40. Dose fractionation (1.25–80 mg/kg/day, every 24, 12, 8, and 6 h) and dose escalation studies (1.25–160 mg/kg, every 24 h) were conducted. The sigmoid E_max_ Hill equation was used to describe the dose-response data. The free-drug plasma *f*AUC_0-24_/MIC ratio was considered the PK/PD index most closely associated with efficacy (R^2^ 0.9268). Median *f*AUC_0-24_/MIC associated with static, 1-log_10_ and 2-log_10_ CFU reduction from baseline were 8.6, 23.2 and 44.4, respectively. Monte Carlo simulation showed 500 mg qd and 750 mg qd oral doses of nemonoxacin were able to achieve 90% probability of target attainment (PTA) against bacteria with MIC of 0.5 mg/L and 1 mg/L. We recommended susceptibility (S) ≤ 0.5 mg/L, intermediate (I) = 1 mg/L and resistant (R) ≥ 2 mg/L as the PK/PD breakpoints for nemonoxacin against *S. pneumoniae*.

## Introduction

Nemonoxacin is a novel nonfluorinated quinolone with potent antibacterial effect against Gram-positive cocci and Gram-negative bacilli, including atypical pathogens penicillin-resistant *Streptococcus pneumoniae* (PRSP) and methicillin-resistant *Staphylococcus aureus* (MRSA) (Adam et al., 2009; Chen et al., 2009; Lauderdale et al., 2010; Li et al., 2010). Nemonoxacin is indicated for community-acquired pneumonia (CAP), and clinical trials demonstrated its good clinical and microbiological efficacy by oral administration of 500 mg once daily for 7 to 10 consecutive days (Guo et al., 2012; Liu et al., 2017; Yuan et al., 2019). This new antibiotic was approved in China in 2016, its approved label gived an initial breakpoint S ≤ 1 mg/L for the definition of susceptible *S. pneumoniae* strains. Breakpoint is used to categorize an organism as susceptible (S), intermediate (I) and resistant (R), which provide a guideline in the rational use of antibiotics in clinical. Setting breakpoints requires three parts of data - epidemiological cut-off, pharmacokinetic/pharmacodynamic (PK/PD) breakpoints and clinical breakpoints. The epidemiological cut-off and tentative clinical breakpoint have been reported recently (http://www.chinets.com/; Jean et al., 2020). PK/PD breakpoints based on *in vivo* PK/PD targets is essential for breakpoints setting, however, it was not available. *In vivo *PK/PD study in an animal infection model has become an important basis for the establishment of PK/PD breakpoints due to its flexible experimental design and similar target results to that in humans (Mouton et al., 2012; Nielsen and Friberg, 2013; Vinks et al., 2014).

*S. pneumoniae* remains the most common cause of CAP despite the usage of vaccines reduced its infection rate (Musher and Thorner, 2014). At the same time, the emergence of bacterial resistance to existing antibiotics such as penicillin, macrolides and tetracycline brought great challenges to CAP treatment (Metcalf et al., 2016; Schroeder et al., 2019). Nemonoxacin provides a new choice for CAP treatment even for resistant bacterial infection.

The objectives of this study were as follows: 1) to describe the PK profiles of nemonoxacin in neutropenic infected mice, 2) to identify the *in vivo *PK/PD index of nemonoxacin associated with the efficacy, and the magnitude of PK/PD index required for various levels of bacterial reduction from baseline, 3) to obtain PK/PD breakpoints of nemonoxacin by performing Monte Carlo simulations using PK data from the human study.

## Materials and Methods

### Bacterial, Media, and Antibiotic

Five clinical *S. pneumoniae* strains [three penicillin-susceptible (PSSP) and two penicillin-intermediate (PISP)] and one reference strain ATCC49619 were used in this study (Table 1). Bacteria were cultured and quantified on a sheep blood agar plate (Shanghai Comagal Microbial Technology Co. Ltd.) and grown for 16 h at 35°C with 5% CO_2_. Nemonoxacin malate (Purity: 73.2%, Lot no: 205MP140803) was supplied by Xinchang Pharmaceutical Factory (Zhejiang, China). The compound was reconstituted and diluted to appropriate concentrations with sterile saline.

**TABLE 1:** *In vitro* activity of nemonoxacin against study organisms.

Organism	Description	Nemonoxacin MIC (mg/L)
ATCC49619	PSSP	0.125
T10222	PSSP	0.125
SP597	PISP	0.125
SP647	PISP	0.125
SPV19031	PSSP	0.25
SPV19032	PSSP	0.25

PSSP, Penicillin-sensitive Streptococcus pneumoniae; PISP, Penicillin-intermediate Streptococcus pneumoniae.

### *In Vitro* Susceptibility Testing

The minimum inhibitory concentration (MIC) of nemonoxacin against all isolates were determined in triplicate with broth microdilution method according to Clinical and Laboratory Institute (CLSI, 2018) guidelines. ATCC 49619 was served as a quality control strain.

### Murine Lung Infection Model

The animal studies were approved by the Experimental Animal Ethics Committee of Pharmacy in Fudan University (2017–01-HSYY-ZJ-01) and followed the Experimental Animal Welfare Review Guide. Six to seven-week-old, specific-pathogen-free, female ICR mice (Sipper-BK, Shanghai, China) weighing 24–26 g were used in all studies. The neutropenic murine lung infection model was established as previously described (Lepak and Andes, 2016; Zhao et al., 2016). Mice were intraperitoneally injected with cyclophosphamide (Sigma) 4 days (150 mg/kg of body weight) and 1 day (100 mg/kg) before lung infection to induce neutropenia. Broth cultures of freshly plated *S. pneumoniae* were grown to logarithmic phase in a 37°C shaker (150 rpm) for about 2 h to a light transmittance of 80% (Industrial and Chemical Measurement, United States). After a 5 times concentration with saline, the bacterial counts of inoculum were 0.5–1 × 10^8^ CFU/ml. Mice were anesthetized with 2, 2, 2 - tribromoethanol (Sigma) and the lung infection were induced by intranasal instillation of 50 μL inoculum of *S. pneumoniae*.

### Pharmacokinetics

Single-dose pharmacokinetics of nemonoxacin was studied in lung infected mice at doses level of 2.5, 10, 40, and 80 mg/kg following subcutaneous administration (0.2 ml/dose). Blood samples were collected at 0.25, 0.5, 1, 2, 4, 6 and 8 h at each dose and bronchoalveolar lavage (BAL) fluid samples were collected in the same mice concomitantly at doses of 2.5, 10 and 80 mg/kg. Three mice were used per time point. Plasma was separated by centrifugation at 4000 *g* for 10 min at 4°C. BAL fluid was obtained using a previously reported method (Berkhout et al., 2015), followed by centrifugation at 4000 *g* for 10 min at 4°C, and the supernatant was collected and stored at −40°C until urea and nemonoxacin concentration analysis.

A liquid chromatography-tandem mass spectrometry (LC-MS/MS) method was used to determine nemonoxacin concentration in plasma and BAL fluid. The lower limits of quantification of nemonoxacin in plasma and BAL fluid were 0.010 mg/L and 0.002 mg/L, respectively. Plasma and BAL urea concentrations were determined using a commercial assay kit (Kehua Bio-Engineering, Shanghai) and were used to correct the epithelial lining fluid (ELF) drug concentration by the method described previously (Keith et al., 2011):

$${\text{drug concentration}}_{\text{ELF}}={\text{drug concentration}}_{\text{BALF}}\times \frac{{\text{Urea concentration}}_{\text{Plasma}}}{{\text{Urea concentration}}_{\text{BALF}}}$$ [1]

The protein binding of nemonoxacin in murine plasma was determined at spiked concentrations of 0.08 mg/L to 0.68 mg/L using an equilibrium dialysis method. In ELF, the drug protein binding was considered negligible.

WinNonlin software (Version 6.3; Pharsight Inc., St. Louis, MO, United States) was employed to calculate the PK parameters using a noncompartmental model, including the elimination half-life (t_1/2_), the area under the concentration-time curve over 24 h (AUC_0-24_), and the peak drug concentration (C_max_). The PK parameter estimation for treatment doses that were not directly determined was calculated based on a compartment model. The ELF penetration ratio was calculated by dividing the ELF AUC_0-24_ value by the plasma AUC_0-24_ value.

### Pharmacokinetic/Pharmacodynamic Index Determination

Neutropenic mice were infected with the standard strain of *S. pneumoniae* ATCC 49619 for a dose fractionation experiment. Treatment with nemonoxacin by the subcutaneous route was initiated 2 h after inoculation. Dose-fractionation study is useful in reducing the interdependence among the PK/PD index and confirming which one is the most important for efficacy. The total daily doses included 1.25, 5, 20, 40 and 80 mg/kg, divided evenly every 6, 8, 12, and 24 h. Groups of three mice were included in each dosing regimen. The mice were sacrificed 24 h after the first dosing of nemonoxacin, and the lungs were aseptically removed and processed for CFU determination (Lepak and Andes, 2016; Zhao et al., 2016). Briefly, the lung tissues were homogenized in 10-fold volumes of 0.9% NaCl under ice bath. Serial 1:10 dilutions of the homogenate were plated overnight at 35°C for CFU counting. The lower limit of viable colony counts was 1000 CFU/ml. Results were expressed as the mean number of log_10_ CFU per gram tissue for three mice. Untreated mice used for the growth control assessment were similarly sacrificed before treatment and 24 h after treatment. Efficacy was calculated as the change in the log_10_ CFU obtained at 24 h for nemonoxacin-treated mice.

To determine the dominant PK/PD index driving efficacy, the number of bacteria in the lung at the end of therapy was correlated with 1) the free drug peak level divided by the MIC (*f*C_max_/MIC), 2) the area under the free concentration-time curve over 24 h divided by the MIC (*f*AUC_0-24_/MIC), and 3) the cumulative percentage of a 24 h period that the free drug concentration in plasma exceeds the MIC (%*f*T > MIC), for each of the dosage regimens studied. The mathematical model used was derived from the Sigmoid E_max_ model:

$$\text{E}={\text{E}}_{0}+\frac{\left({\text{E}}_{\text{max}}-{\text{E}}_{0}\right)\times {\text{C}}^{\gamma }}{{\text{EC}}_{50}^{\gamma }+{\text{C}}^{\gamma }}$$ [2]

where E is the effect, in this case, the log_10_ change in CFU per gram lung between treated mice and untreated controls after the 24-h period of study, E_max_ is the maximum effect, C is the PK/PD index value, EC_50_ is the value of PK/PD index required to achieve 50% of the E_max_, and *γ* is the slope of the dose-effect curve. The *R* ^2^ value from non-linear regression analysis (WinNonlin 6.3) was used to assess the correlation of treatment efficacy with each of the PK/PD indices.

### PK/PD Targets for Efficacy

Dose-ranging efficacy studies to determine the PK/PD targets for net stasis, 1-log_10_CFU and 2-log_10_CFU kill were then performed with five clinical *S. pneumoniae* strains using the murine neutropenic lung infection model. 2- or 4-fold-increasing single dosing regimens of nemonoxacin were administered with doses varied from 1.25 to 160 mg/kg. Groups of three mice were included per dose level. Treatment was initiated 2 h after inoculation. Animals were sacrificed at 24 h after therapy, and the lungs were aseptically removed and immediately processed for CFU determination. A sigmoid dose-response model derived from the Hill equation was used to calculate the dose, and PK/PD targets of nemonoxacin producing a net bacteriostatic effect, 1-log_10_ kill and 2-log_10_ kill over 24 h compared to the organism burden at the start of treatment.

### Monte Carlo Simulation

Monte Carlo simulations were performed to evaluate the oral dosing regimens for humans. The simulated data of the AUC_0-24_ were obtained based on logarithmic normal distribution using PK parameters of nemonoxacin in healthy Chinses subjects from a phase I clinical trial (Guo et al., 2012). The protein binding of nemonoxacin in human plasma is 16% (Lin et al., 2010). The MIC distribution of 46 *S. pneumoniae* strains was obtained from Wu’s study (Wu et al., 2015), and the MIC data used for CFR analysis were generated based on discrete distribution according to the fractions of the isolates at each MIC level. The probability of target attainment (PTA) and the cumulative fraction of response (CFR) of 500 mg qd or 750 mg qd dosing regimens of oral nemonoxacin was calculated, using the *in vivo *PK/PD targets obtained from our study. The simulation was performed 5000 times with Matlab software version R2018b (MathWorks, Inc., United States) (Wu et al., 2015). The MIC values with PTA reaching 90% were considered as PK/PD breakpoints of nemonoxacin.

## Results

### *In Vitro* Susceptibility Testing

The MIC values of nemonoxacin against six *S. pneumoniae* isolates used in this study are shown in Table 1, ranging from 0.125 to 0.25 mg/L.

### Pharmacokinetics

**FIGURE 1:**
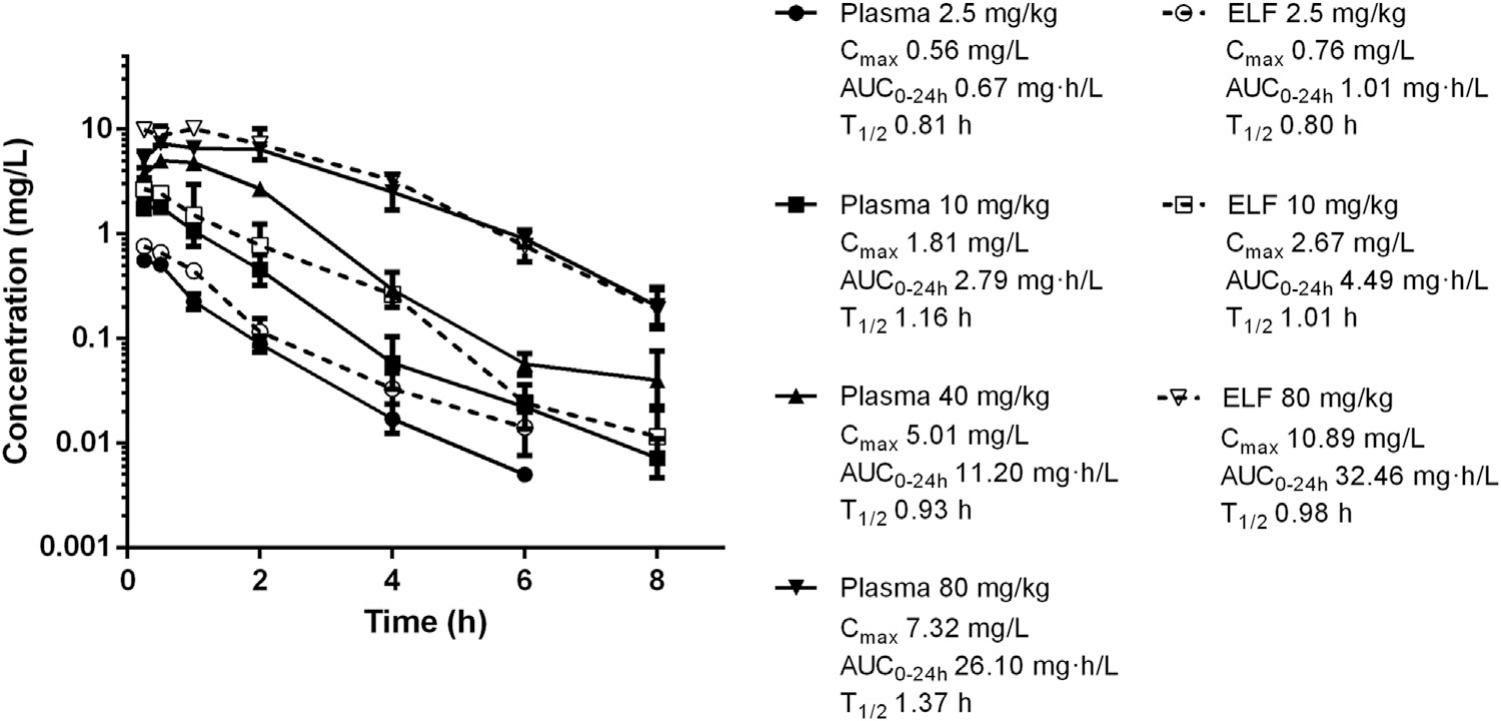
Pharmacokinetics of nemonoxacin in plasma and epithelial lining fluid (ELF) following single subcutaneous doses at 2.5–80 mg/kg in neutropenic lung infected mice. Plasma was sampled at doses of 2.5, 10, 40 and 80 mg/kg. ELF was studied at doses of 2.5, 10 and 80 mg/kg. Groups of three mice were sampled for each time point. Each symbol represents the mean value of three mice. The error bar represents the standard deviations. Pharmacokinetic parameters including maximum drug concentrations (C_max_), the AUC from 0 to 24 h (AUC_0-24_), and elimination half-life (T_1/2_) for each dose.

Single-dose PK of nemonoxacin in plasma and ELF are shown in Figure 1. The elimination half-life in plasma ranged from 0.81 to 1.37 h. C_max_ concentrations ranged from 0.56 to 7.32 mg/L. AUC_0-24_ values ranged from 0.67 to 26.10 mg/L and were linear across 2.5–80 mg/kg dose range (*R* ^2^ > 0.99). The plasma PK of nemonoxacin was well described by a two-compartment model. Based on the total AUC and C_max_ ratio in ELF and plasma, the penetration of nemonoxacin into ELF were 1.45 ± 0.19 and 1.40 ± 0.06, respectively. The protein binding of nemonoxacin in murine plasma varied from 34 to 41% in the range of 0.08–0.68 mg/L, with a mean binding rate of 39%. The penetration ratio of the free-drug into ELF based on AUC and C_max_ were 2.30 ± 0.10 and 2.39 ± 0.32, respectively, which further confirmed a considerable pulmonary distribution of nemonoxacin.

### PK/PD Index Determination

**FIGURE 2:**
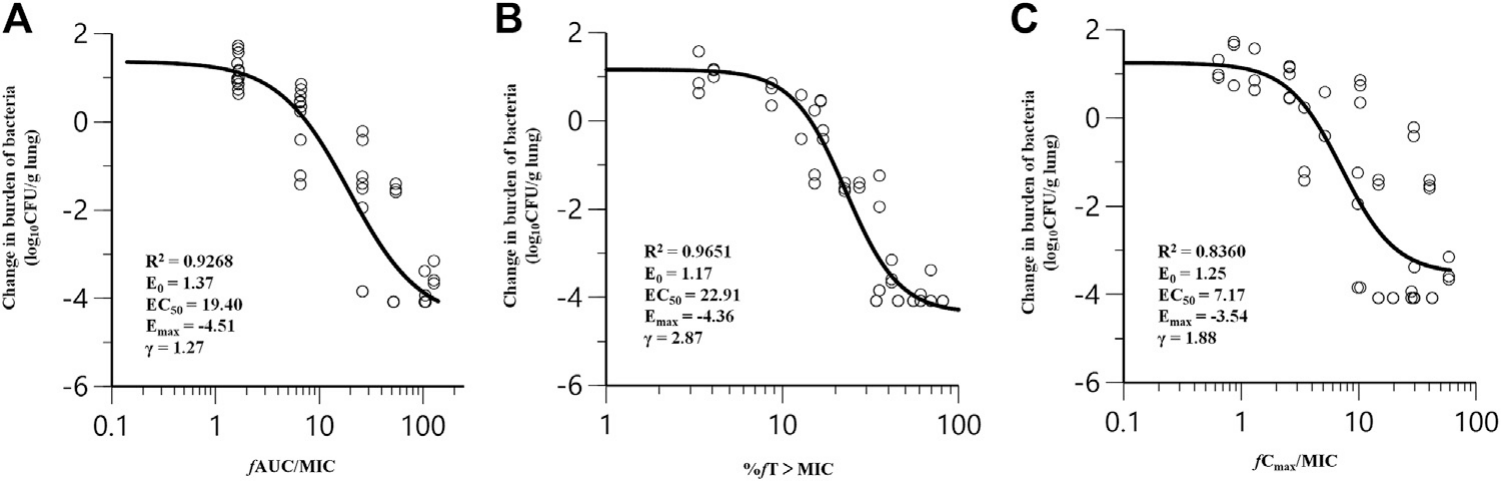
Correlation of pharmacokinetic/pharmacodynamic (PK/PD) indices *f*AUC_0-24_/MIC **(A)**, %*f*T > MIC **(B)** and *f*C_max_/MIC **(C)** with efficacy for nemonoxacin in a neutropenic murine lung infection model caused by *Streptococcus pneumoniae* ATCC49619 in dose-fractionation study. Treatment was initiated at 2 h postinfection. Nemonoxacin was subcutaneously administered with a dosing range of 1.25–80 mg/kg, in once daily (q24h), twice daily (q12h), three times a day (q8h) and four times a day (q6h). Each circle represents data for each mouse. *R* ^2^, the square of the correlation coefficient.

Nemonoxacin treatment produced 4.08 log_10_ reduction of CFU burden against ATCC49619 in the dose fractionation experiment. The dose-response curves with different dosing intervals showed the bactericidal effect was improved with the increase of dose and the decrease of the dosing interval. The relationship between efficacy and three PK/PD indices *f*AUC_0-24_/MIC, %*f*T > MIC and *f*C_max_/MIC are shown in Figure 2, and the value of the square of the correlation coefficient (*R* ^2^) were 0.9268, 0.9651 and 0.8360, respectively. Both of the %*f*T > MIC and *f*AUC_0-24_/MIC correlated well with efficacy.

Nemonoxacin exerts antimicrobial effects by inhibiting bacterial DNA replication, similar to other fluoroquinolones. The half-life of nemonoxacin in mice is short, about 1/10 of that in humans, which may cause regrowth due to insufficient concentration, increasing the correlation of %*f*T > MIC with efficacy. In clinical application, *f*AUC/MIC is easier to estimate accurately. Considering all the above, we used *f*AUC_0-24_/MIC as the PK/PD index in case of close *R* ^2^ values in this study.

### PK/PD Targets for Efficacy

**FIGURE 3:**
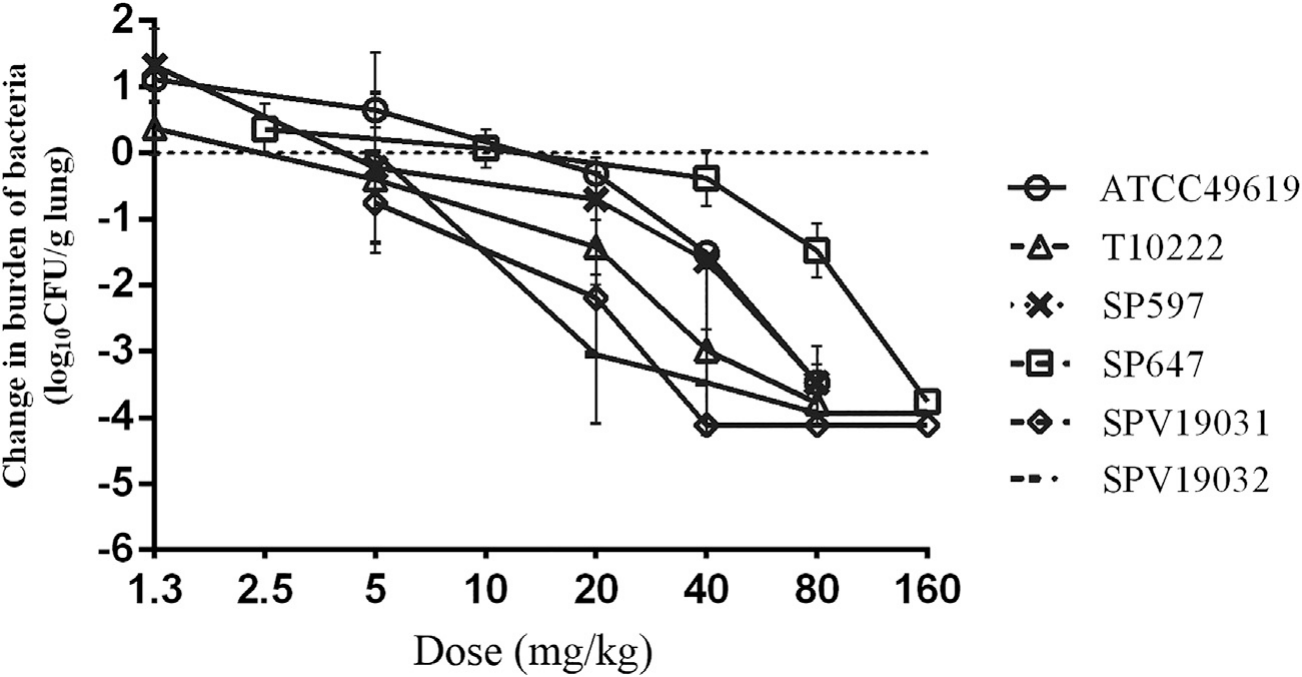
*In vivo* dose-response curves for nemonoxacin against six *Streptococcus pneumoniae* strains over a 24 h study period after a single dose administration in the neutropenic murine lung infection model. Each symbol represents the mean and standard deviation from three mice. The burden of organisms was measured at the start and end of therapy. The horizontal dashed line at 0 represents no net change from baseline.

**FIGURE 4:**
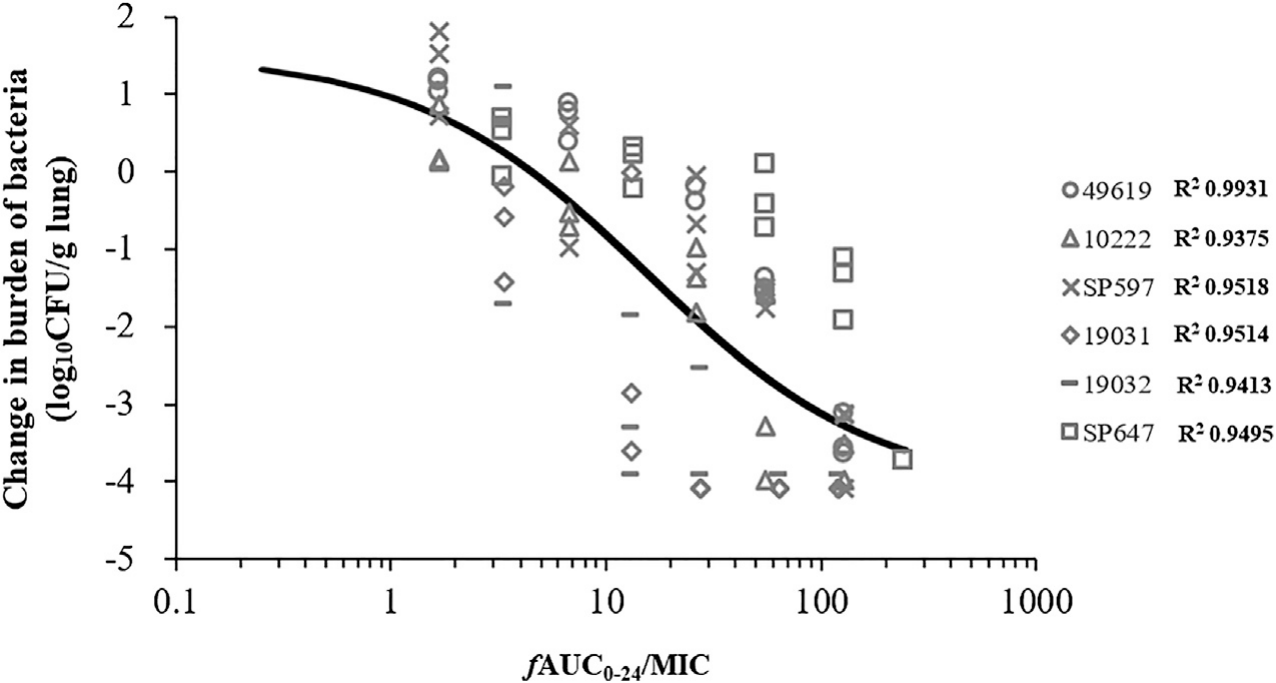
Correlation between PK/PD index *f*AUC_0-24_/MIC and efficacy of nemonoxacin against pooled data of six *Streptococcus pneumoniae* strains using a neutropenic murine lung infection model in dose-escalation experiment. Treatment was initiated at 2 h postinfection. Nemonoxacin was subcutaneously administered with single dose range of 1.25–160 mg/kg. Each point represents data for each mouse. *R* ^2^, square of the correlation coefficient.

Five additional *S. pneumoniae* strains were used in the dose-escalation experiment to determine the PK/PD targets required for efficacy. The dose-response data for each of the strains are shown in Figure 3. Nemonoxacin demonstrated potent efficacy against the *S. pneumoniae* strains in our study*.* The maximal effect varied from 3.0 to 3.6 log_10_ CFU kill compared with the initial bacterial burden. The dose-response data were modeled using the sigmoid E_max_ equation, showing *f*AUC_0-24_/MIC was a strong predictor of treatment outcomes base on regression analysis (Figure 4, *R* ^2^ 0.8311). The doses, plasma free and ELF total AUC_0-24_/MIC values necessary to produce a static, 1-log_10_ kill and 2-log_10_ kill effect in the bacterial burden are shown in Table 2. The median plasma *f*AUC_0-24_/MIC and ELF AUC_0-24_/MIC targets needed for 2-log_10_ kill were 44.4 and 105.6, respectively.

**TABLE 2:** *In vivo* activity and PK/PD analysis of nemonoxacin against study organisms.

Strains	Bacterial burden at start of therapy (log_10_ CFU/lung)	Growth in controls at 24 h (log_10_ CFU/lung)	ΔLog_10_CFU = 0			ΔLog_10_CFU = −1			ΔLog_10_CFU = −2			
			Dose (mg/kg)	*f*AUC_0-24_/MIC target		Dose (mg/kg)	*f*AUC_0-24_/MICTarget		Dose (mg/kg)	*f*AUC_0-24_/MIC Target	
				Plasma	ELF		Plasma	ELF		Plasma	ELF
ATCC49619	6.85	0.88	15.6	20.2	48.0	30.6	41.5	98.7	47.9	68.3	162.4
T10222	7.01	0.23	3.3	4.5	10.6	12.7	16.8	39.9		25.1	33.1	78.7	
SP597	7.11	1.87	10.6	12.6	29.9	22.8	29.5	70.2	41.1	55.7	132.4	
SP647	6.76	1.86	24.6	37.1	88.3	53.3	76.7	182.3	88.1	127.2	302.5	
SPV19031	7.51	1.50	3.2	2.1	5.1	6.6	4.4	10.4	12.2	8.3	19.7	
SPV19032	7.33	1.35	5.0	3.3	7.8	7.8	5.2	12.3	12.0	7.9	18.7	
Mean			10.4	13.3	31.6	22.3	29.0	69.0	37.7	50.1	119.1	
Median			7.8	8.6	20.3	17.8	23.2	55.1	33.1	44.4	105.6	
SD			8.5	13.5	32.2	17.8	27.4	65.1	28.7	45.0	107.0	

### Monte Carlo Simulation

**FIGURE 5:**
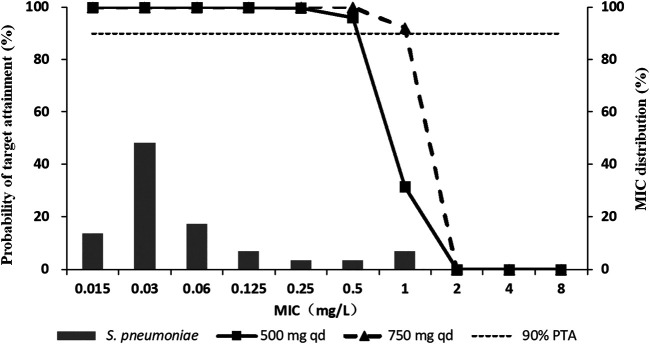
Probability of target attainment (PTA) of nemonoxacin in terms of *f*AUC_0-24_/MIC ≥44.4 (2-log_10_ CFU kill) following oral administration of nemonoxacin malate sodium chloride in healthy Chinese subjects, overlaid on the MIC distribution for *S. pneumoniae*.

Monte Carlo simulation indicated that the CFR of nemonoxacin for 500 mg qd and 750 mg qd achieved 98% or higher against *S. pneumoniae* in terms of the *f*AUC_0-24_/MIC ≥44.4. The PTA results of the two dosing regimens of nemonoxacin are shown in Figure 5. For strains with MIC ≤0.5 mg/L, the PTA of 500 mg qd continuous dosing regimen reached 95%, and nearly 100% when the dose increased to 750 mg. For strains with MIC ≤1 mg/L, PTA was 32% for 500 mg and increased to 92% for 750 mg. The MIC values corresponding to the two dosing regimens were 0.5 mg/L and 1 mg/L when PTA reached 90%, respectively. The PK/PD breakpoint values of S ≤ 0.5 mg/L, I = 1 mg/L, and R ≥ 2 mg/L for nemonoxacin against *S. pneumoniae* were recommended.

## Discussion

Using a neutropenic murine lung infection model, our study determined the magnitudes of *f*AUC_0-24_/MIC of nemonoxacin associated with various levels of bacterial reduction for *S. pneumoniae* strains. And the clinical dosing regimens were evaluated based on the targets, where we also get the PK/PD breakpoints.

The inclusion of multiple bacterial isolates with various susceptibility should be considered in animal PK/PD experiment design to obtain a robust PK/PD target (Jürgen et al., 2019). One ATCC and five clinical strains of *S. pneumoniae* with MICs ranging from 0.125 mg/L to 0.25 mg/L were used in this study. We didn’t get a strain with higher MIC because these strains were unable to grow stably in the murine lungs in the preliminary experiment. A MIC of 0.125 mg/L can also be 0.25 or 0.06 mg/L, accordingly this study was based on the same MIC or in the best case scenario on two different MICs, which is a limitation. However, it should be noted that the MIC values of the selected strains were equal to MIC_50_ (0.125 mg/L) and MIC_90_ (0.25 mg/L) of nemonoxacin against *S. pneumoniae* according to *in vitro* study (Zhu et al., 2015). Besides, PSSP and PISP were included in our study which indicated the weak impact of penicillin resistance on nemonoxacin activity. There was a difference in the bactericidal effect of nemonoxacin while the MIC values of the strains were similar, it may be caused by the differences in virulence, tolerance and adaptive capacity to hosts between the strains. It also illustrates the importance to adopt a PK/PD approach combining *in vivo* and *in vitro* studies for dosing regimen evaluation rather than based on MIC values alone.

The PK/PD indices of quinolone antibiotics are generally *f*AUC/MIC and *f*C_max_/MIC (Ambrose et al., 2001; Ambrose et al., 2003), while in this study, E_max_ model analysis showed that for nemonoxacin, both *f*AUC_0-24_/MIC and %*f*T > MIC correlated well with efficacy. We considered it is the short half-life of the drug in mice that caused the increase of the correlation between %*f*T > MIC and antibacterial effect. The efficacy of an antimicrobial agent is determined by both the killing rate and the sustained bactericidal time (Tam and Nikolaou, 2011). The killing rate is concentration-dependent, while the bactericidal duration is related to the half-life and the post-antibiotic effect (PAE) of the drug. For concentration-dependent drugs with short half-life, both increasing the drug concentration (by increasing the dose) and prolonging the exposure duration (by decreasing the dosing interval or prolonging the infusion time) could improve efficacy, which shows both AUC/MIC and %T > MIC are PK/PD drivers. If the drug has a long half-life and PAE, since %T > MIC is easy to reach a high level, increasing the drug concentration could improve the effect, which mainly shows the concentration-dependent characteristics. Animal PK/PD studies of penicillin and amikacin, as well as some *in vitro* studies, showed an increased correlation of antibacterial effect with *f*AUC/MIC when the half-lives of the drug were prolonged, and a shortened half-life is associated with %*f*T > MIC better (Craig et al., 1991; Nielsen et al., 2011). Tam’s study also showed there could be different PK/PD characteristics at different dose levels for the same drug (Tam and Nikolaou, 2011).

The clearance of the drug in mice is much faster than that in humans (Zhao et al., 2016), therefore, the effect of half-life should be particularly considered when the dose fractionation experiment in the murine infection model was used to determine the PK/PD index. Renal impairment mice or continuous dosing systems could be used to mimic human PK. The half-life of nemonoxacin in humans is about 11 h (Wu et al., 2015). With the dosing regimens of 500 mg qd and 750 mg qd, its %T > MIC_90_ (0.25 mg/L) is close to 100%. Combined with our E_max_ analysis results, the *f*AUC_0-24_/MIC is determined to be the PK/PD index.

Q24h dosing interval may result in inadequate plasma concentration due to the short half-life of the drug, and bacterial regrowth occurred (Bulik et al., 2017). We also performed E_max_ model fitting after removing the q24h data, and the results still showed closed *R* ^2^ values of *f*AUC_0-24_/MIC and %*f*T > MIC (0.9655 and 0.9603). It also suggested that nemonoxacin may have a more complicated bactericidal pattern that is both concentration-dependent and time-dependent. The *in vitro *PK/PD study of nemonoxacin showed dualism in the antibacterial effect and suggested a proper split of daily dose for strains with higher MICs (Liang et al., 2013). Our results indicated that this dualism phenomenon may also exist *in vivo* for strains with lower MICs.

The target value of *f*AUC_0-24_/MIC of nemonoxacin against *S. pneumoniae* from *in vitro *PK/PD study was 47.05 (Liang et al., 2013), and the *in vivo* target value of quinolones to achieve antibacterial effect against *S. pneumoniae* was 25–50 (Ambrose et al., 2001; Ambrose et al., 2003). In this study, the *f*AUC_0-24_/MIC target of nemonoxacin required for 2-log_10_ kill effect against *S. pneumoniae* in animals was 44.4, which was consistent with the *in vitro* results. Considering the effect of immune clearance *in vivo* and the q24h dosing interval, the actual magnitude of the target may be lower than 44.4, so 44.4 could be regarded as a relatively conservative result. Besides, the *f*AUC_0-24_/MIC targets were obtained based on PK/PD studies for strains with lower MICs, and with the development of drug resistance, it is still necessary to screen strains with higher MICs and different resistance mechanisms for PK/PD studies to obtain robust targets.

The PK/PD breakpoints based on the clinical dosing regimens of nemonoxacin were S ≤ 0.5 mg/L, I = 1 mg/L, and R ≥ 2 mg/L. According to CHINET data, the *in vitro* epidemiological cut-off of nemonoxacin are wild type (WT) ≤ 0.5 mg/L and non-wild type (NWT) ≥ 1 mg/L (http://www.chinets.com/). In clinical application, the cure rate of nemonoxacin in phase II and III clinical studies were 85–90% (Liu et al., 2017; Yuan et al., 2019), there were few treatment failure samples so that we can’t obtain effective PK/PD target values just from the clinical data. Taken all above together, S ≤ 0.5 mg/L, I = 1 mg/L, and R ≥ 2 mg/L were recommended as breakpoints of nemonoxacin.

The recommended dose from oral nemonoxacin label is 500 mg qd. A phase II clinical study of nemonoxacin showed the incidence of adverse events (AE) were 30.6 and 35.6% for 500 and 750 mg, respectively. All of the AEs were mild or moderate, including loss of appetite, nausea, vomiting and stomach discomfort, and were tolerable to patients (Liu et al., 2017). A thorough QT clinical study suggested that nemonoxacin 500 mg oral dose had no risk of QTc prolongation (ΔΔQTc 8.1 s) and 750 mg had a potential risk of prolongation (ΔΔQTc 10.4 s) (Zhao et al., 2018). Considering the incidence of adverse reactions in phase II clinical trial and the risk of QTc prolongation, 500 mg was selected as the phase III clinical trial dose, the incidence of drug-related AEs was 19.4% (Yuan et al., 2019). 500 mg dose regimen has confirmatory clinical trial evidence and slightly better safety than 750 mg, which could be first recommended for the treatment of patients with bacterial infections. Our PK/PD analysis suggests that for infections caused by strains with higher MICs (MIC = 1 mg/L), or patients who do not improve with 500 mg therapy and have a suspected or pathogen diagnosis of PRSP infection, 750 mg qd dose regimen could be used.

## Conclusion

Nemonoxacin showed a potent bactericidal effect against *S. pneumoniae* utilized in this study, The plasma *f*AUC_0-24_/MIC of 44.4 was required for achieving 2-log_10_ kill effect in mice lungs. In terms of this target, PTA analysis demonstrated good clinical efficacy with nemonoxacin dose regimens of 500 mg qd and 750 mg qd. We recommended S ≤ 0.5 mg/L, I = 1 mg/L, and R ≥ 2 mg/L as the PK/PD breakpoints of nemonoxacin against *S. pneumoniae*, which could provide a basis for dosing regimen evaluation and facilitate the rational use of the drug in the clinical setting.

## Data Availability

The raw data supporting the conclusions of this article will be made available by the authors, without undue reservation.
